# Global bioethics – myth or reality?

**DOI:** 10.1186/1472-6939-7-10

**Published:** 2006-09-11

**Authors:** Søren Holm, Bryn Williams-Jones

**Affiliations:** 1 Cardiff Institute of Society, Health and Ethics, Schools of Law and Social Sciences, 53 Park Place, Cardiff University, Cardiff, Wales, CF10 3AT, United Kingdom; 2 Section for Medical Ethics, University of Oslo, PO Box 1130 Blindern, N-0318 Oslo, Norway; 3 Groupe de recherche en bioéthique & Département de médicine sociale et préventive, Université de Montréal, C.P. 6128, succ. centre-ville, Montréal (Québec) H3C 3J7, Canada

## Abstract

**Background:**

There has been debate on whether a global or unified field of bioethics exists. If bioethics is a unified global field, or at the very least a closely shared way of thinking, then we should expect bioethicists to behave the same way in their academic activities anywhere in the world. This paper investigates whether there is a 'global bioethics' in the sense of a unified academic community.

**Methods:**

To address this question, we study the web-linking patterns of bioethics institutions, the citation patterns of bioethics papers and the buying patterns of bioethics books.

**Results:**

All three analyses indicate that there are geographical and institutional differences in the academic behavior of bioethicists and bioethics institutions.

**Conclusion:**

These exploratory studies support the position that there is no unified global field of bioethics. This is a problem if the only reason is parochialism. But these regional differences are probably of less concern if one notices that bioethics comes in many not always mutually understandable dialects.

## Background

The term 'global bioethics' has been bandied about in recent years, although its precise meaning has often been unclear. For some, it has been a call to globalize the concerns of bioethics by focusing more attention on, for example, issues for resource poor countries, public health, or global justice and equity. For others, global bioethics has been a statement about the right way to pursue bioethics, that there is one global set of principles. For a third group, which will be the focus of our analysis, it has been a statement of final achievement, that bioethics has become a global field of inquiry. In this paper, we use a sociological approach to highlight the connections or lack thereof between bioethics researchers and research centers as a proxy for examining the ways in which bioethics scholarship is 'performed' around the world.

We are clearly not the first to question whether there really is such a thing as a global or unified field of bioethics. In a clash of the titans, Al Jonsen and Alistair Campbell have been in dispute over the history of bioethics, with Campbell [1] claiming that Jonsen's [2] unified history is only possible by neglecting developments outside of the US; for another example of this neglect see Baker's review [3] of Walter and Klein [4]. More generally, and particularly following the publication of David Rothman's seminal book *Strangers at the Bedside *[5], there has been a vigorous debate in recent years (much of it coming from social scientists) about the drivers behind the development of bioethics, how these have shaped the field and their effects on the global dissemination of bioethics [6,7].

There has also been a long-standing debate concerning whether the principles and methods of American bioethics are the same as the principles and methods of European, Asian or African Bioethics. There is, for instance, a large literature examining whether the four principles proposed in successive editions of Beauchamp and Childress' *Principles of Biomedical Ethics *are universally valued or irredeemably American, as well as some suggestions for specific regional or cultural reformulations of these four principles [8-22]. Since we do no want to repeat these earlier debates, we have decided not to focus on the *arguments *used in bioethics discourse. Instead, by paying attention to what might be called the 'publicly observable behavior' within the field, we aim to investigate whether bioethicists in different regions of the world *behave *in the same way in their academic activities. We hope thus to provide a small contribution to the project identified by De Vries in 2003, that " [t]wo features of the bioethical project merit the immediate attention of sociologists of bioethics: the *form *and *substance *of the field" [23].

If bioethics is a unified global field, or at the very least a closely shared way of thinking, then we should expect bioethicists to behave the same way in their academic activities anywhere in the world. There is obviously great diversity in any academic field of study, with specializations and associated literatures, regional and linguistic differences, contested domains, etc. But established academic disciplines such as philosophy, sociology, medicine, or engineering arguably have common academic languages, agreed upon canonical texts, and facts and values that are no longer in dispute. To some extent, then, these disciplines, and in particular their subspecialty fields of inquiry, will be 'global' in nature (note that the study of, for instance, 'Chinese philosophy' is not restricted to China or to Chinese philosophers). If bioethics is thought to be similarly unified, then a Danish bioethicist should generally be interested in the same topics, read the same books, attend the same conferences, cite the same authors, look at the same websites, and work in the same way as an American or Canadian bioethicist. Geography, language and local regulatory environment would clearly matter to some of these activities, but in general the research questions, methods and modes of analysis would be expected to be quite similar. In this paper, we are interested in investigating whether this uniformity of behavior assumption is true across a range of behaviors that are amenable to sociological analysis.

We feel that we have some specific personal qualifications for this task. To a certain extent, we have each followed one of the 'traditional' American paths into the field of bioethics: Holm completed an MD, a few years clinical practice and then did a PhD in medical ethics; Williams-Jones majored in philosophy, then completed a bioethics Masters specialization and an interdisciplinary applied ethics PhD. Yet both of us are also natives of countries (Denmark and Canada respectively) outside the traditional US and UK heartlands of bioethics, and both have experienced the transition (not to mention the intellectual and practical differences) involved in moving to another country (the UK), ostensibly to continue our careers within the unified, global field of bioethics. Finally, while having entered the field through relatively traditional paths, we are both interdisciplinary thinkers who are interested in integrating empirical methods and social science critiques into our bioethical theorizing.

Because of our own linguistic (in)abilities, we have restricted our research to English language bioethics; because of our disciplinary backgrounds, we have focused on medical ethics, which we understand as an ethical analysis directed primarily at those issues arising in the medical context; and because of our academic training, we have employed research tools amenable to those not trained in the social sciences. The first restriction in the scope of our analysis is clearly unfortunate, but understandable given the small scale of our study – a comprehensive multilingual review would make an interesting large-scale project! The second restriction is perhaps a slight bias, but we believe a legitimate one given the historical dominance of medical ethics (and research ethics) in bioethics scholarship. As for our third restriction, we acknowledge that a detailed sociology of bioethics is clearly desirable, but suggest that our limited empirical study can still provide a useful contribution to understanding the developing and diverse nature of the field of bioethics.

### Research question

Is bioethics a unified global field of inquiry in a sociological sense, i.e., not in terms of arguments, but in terms of connections between researchers and research centers?

The field of bioethics is sometimes conceived of as a homogeneous 'American' enterprise that has been exported and accepted (imposed) worldwide. Worse yet, the field is often critiqued as if it were static (rooted in respect for autonomy and the 'Four Principles' approach of the 1980s), unreflective and uninformed by, for example, developments in feminist theory, AIDS activism, ELSI and the Human Genome Project, public health ethics or critiques from the social sciences [24,25].

A moment's reflection, however, shows the falsity of the homogeneity thesis. To begin with, the very term 'bioethics' is used to describe a diverse set of research areas such as, but not limited to:

• biomedical ethics: general term, sometimes synonymous with bioethics

• medical ethics: ethics in relation to the practice of medicine

• clinical ethics: specific ethical issues in the hospital/clinical context; overlap with medical ethics

• research ethics: ethical issues in human and animal subjects research

• health care ethics: including medical, clinical and research ethics, synonymous with bioethics, but less focus on the 'bio' and more on health care

• biotech ethics/genethics/ELSI/ELSA/GE^3^LS: specific focus on genetics and biotech issues, not necessarily about hospitals or clinical care

• public health ethics: health care systems, public health issues, policy

• neuroethics: ethics in pharma/psychotherapy, brain imaging, etc.

• administrative ethics: ethics of hospital and health systems functioning – type of applied ethics, part of bioethics although not prominent

• professional ethics: another applied ethics, focus in bioethics context on medical professionals, lawyers, etc.

Nor is the 'bio' part of the 'bioethics' uncontested; some scholars see it as inappropriately focusing a diverse field of inquiry on the biological and/or genetic aspects, which has lead to the use by some scholars and practitioners of other labels such as 'biomedical ethics' or ' health care ethics'.

While referring to a field of study or topic of academic research, the term 'bioethics' is also a professional designation, i.e., 'bioethicist', given to those individuals engaged in ethics consultations. These include but are not limited to: clinical ethicists or members of ethics committees, ethicists providing government and policy advise or serving on statutory committees, ethicists engaged in public outreach or news media commentating, etc. Many of these ethicists will also have academic affiliations, but some – particularly in the US – are private consultants.

The formal or informal professionalization of the field, however, is also a source of contention. Some have argued that a core set of competencies and a basic knowledge base is essential for bioethics consultation, but that consultation can nonetheless be performed by scholars from a variety of disciplines and practiced in diverse professional settings [26,27]. Others see bioethics as a field in need of formal professional accreditation and regulation, codes of ethics, etc. as would be the case for members of the various medical professions [28,29].

Despite the rapid growth of bioethics research centers and graduate programs (primarily in the US), there are still relatively few places where one can obtain a formal bioethics degree, especially at the level of the PhD – people trained and working in the field tend to come from and have formal accreditation in disciplines such as philosophy, law, medicine, nursing, the social sciences, and a few interdisciplinary programs. Thus many people who write on issues broadly understood as 'bioethics' are philosophers, lawyers, sociologists, economists, etc. who would not consider themselves and in fact might even strongly resist being called 'bioethicists' [30].

Some of this resistance to the label has, in recent years, had much to do with bioethics getting a 'dirty name' in the US. Bioethicists there are perceived by some scholars to be 'selling out' to industry through the acceptance of (sometimes) lucrative consulting fees [31,32], or by actively courting industry funding for their academic research centers [33]. In the UK, with its more restricted history of professionalization of bioethics (e.g., few hospitals have clinical ethicists on staff) the label is rejected by many scholars because of its association with an 'old fashioned' and largely philosophical and medical approach to theorizing and problem solving in the clinical context. In Canada, the term and its professional label appear to be less contentious; for example, the main scholarly association is the Canadian Bioethics Society (CBS). Nevertheless, there has been strong resistance in Canada towards calls for greater professionalization and formal accreditation of those practicing in the field, but at the same time, a recognition of the need for improved peer and institutional support for practicing bioethicists (e.g., the CBS has established a Working Group on Conditions for Bioethics in Canada [34]).

Finally, a more general critique of the field, and one that is coming both from within and outside (largely from social scientists), is a challenge to the moral authority being given to or taken on by some bioethicists, particularly as evidenced in the prominence of bioethics in the media and the high profile of many government ethics advisory groups. The contention is that bioethicists, or those scholars who reject the term but still work in the field, should refrain from being treated as or acting like the 'New Priests' of contemporary secular society and public discourse. Instead of, or in conjunction with, providing 'answers' to the social, ethical and political issues associated with new biomedical technologies, bioethicists should be asking tough questions about, for example, government and industry involvement in biomedicine, the continued institutionalization of inequity in access to health care and other social services, or global (in)justice [35]. Far from being a unified profession, discipline or field of study, bioethics is arguably a field in turmoil – some would even argue that it is in crisis and in need of radical reformulation [36].

If we find that bioethics is not a unified global field in a sociological sense, then some of the critical voices mentioned above would probably feel vindicated. But maybe such a finding should be seen as a sign of hope and not of crisis? Whatever the critics might say about one universal, global or American bioethics, it is our view that there are other kinds of bioethics out there.

## Methods

Following the lead of De Vries [23], Haimes [37] and López [38], sociologists who are exploring the interface between theoretical ethics and empirical social science research, we have in this paper employed empirical tools to analyze three behaviors, ranging from the strictly institutional to the strictly private. Specifically, we examine:

1. The web-linking patterns of bioethics institutions;

2. The citation patterns of bioethics papers; and

3. The buying patterns of bioethics books.

Each of these analyses is described in detail below (in the Results section). They all fall within the category of exploratory analyses (i.e., 'quick and dirty'), mainly because they rely on easily accessible public data that is amenable to computerized analysis. Each could be pursued more exhaustively and developed into its own project that would, for instance give us the definitive account of citation patterns in bioethics from the inception of the discipline to the present day. The intent here, however, is not to be comprehensive or all inclusive, but to provide a 'snapshot in time' that is sufficient to answer the question of our article, i.e., is there a global bioethics in a sociological sense. Each of the analyses can give only an indication of whether there is one global field of bioethics, and if so, what its structure and organization might be; but if they all point in the same direction, then they may provide us with something slightly stronger than an indication, because they look at three different kinds of behaviors. Combined, they might even count as evidence.

## Results

### The web-linking patterns of bioethics institutions

A web-presence is an increasingly important feature of the communications strategy of bioethics organizations, whether they be academic (e.g., research centers, departments), official (e.g., national bioethics commissions), or non-governmental (e.g., community or interest groups). Most of these websites link to other sites and although we would not claim that all decisions about who to link to are made after long and detailed consideration, there is no doubt that web-linking is not random [39,40]. It is therefore of interest to study who links to whom in the bioethics sphere. Most analyses of web-linking in general shows clustering of some kind; there are clusters of websites that are closely linked together, often by reciprocal in- and out-links, whereas links between clusters are more sparse and most sites in one cluster will not link to any sites in another cluster. Clustering can therefore be expected in the bioethics field as well and the interesting issue is whether the clusters are to some degree interpretable, i.e., whether it is possible to infer what underlies or explains the web-linking behavior that creates the clusters.

In order to perform such an analysis, we have used the IssueCrawler web-cartography crawler developed by the GovCom.org Foundation in Amsterdam [41]. When provided with a starting list of websites, IssueCrawler will search these websites and identify all out-links (i.e., all other sites that the sites on the original list link to). It will then, given the settings we provided for the crawl, retain the websites on the original list and add all websites receiving at least two in-links in the first crawl; these will be deemed to be part of the initial issue network since they have, so to speak, been validated by at least two of the original sites as being relevant. The crawler will then perform a second crawl with the new larger list as the base and deliver a map of all websites receiving at least two in-links in the second crawl. This means that the original sites are not on the final mapping if other sites do not link to them. This result is then mapped according to either the number of in-links to a site or the total number of links taking account of both in- and out-links. Closely linked sites are grouped together and the size of the circle representing a site on the map corresponds to the number of links.

Our starting links were derived from an initial long list containing: 1) the 20 top Google sites for each of the search words 'bioethics', 'bioethik', 'bioetik', 'bioetica' and 'bioethique', and 2) sites identified by colleagues in Germany, France, Italy and Spain who were asked to provide sites with good link lists. From the long list we excluded sites with very limited link lists and aimed to have roughly 5–10 sites in each category of our list to ensure reasonable geographical coverage (France, Germany, the United Kingdom, Italy and Spain, and the United States), and coverage of both official and academic bioethics. Some websites were placed into a 'Christian' category, although they could as well have been included in one of the other categories according to language and geography; the intent of categorizing by religion was to address the perception by some that there is a growing disconnect between secular and Christian bioethics (see Additional file 1). In order to be able to detect the effects of such a possible disconnect on web linking it was important to ensure that there were both secular and Christian sites in the starting list.

**Figure 1:**
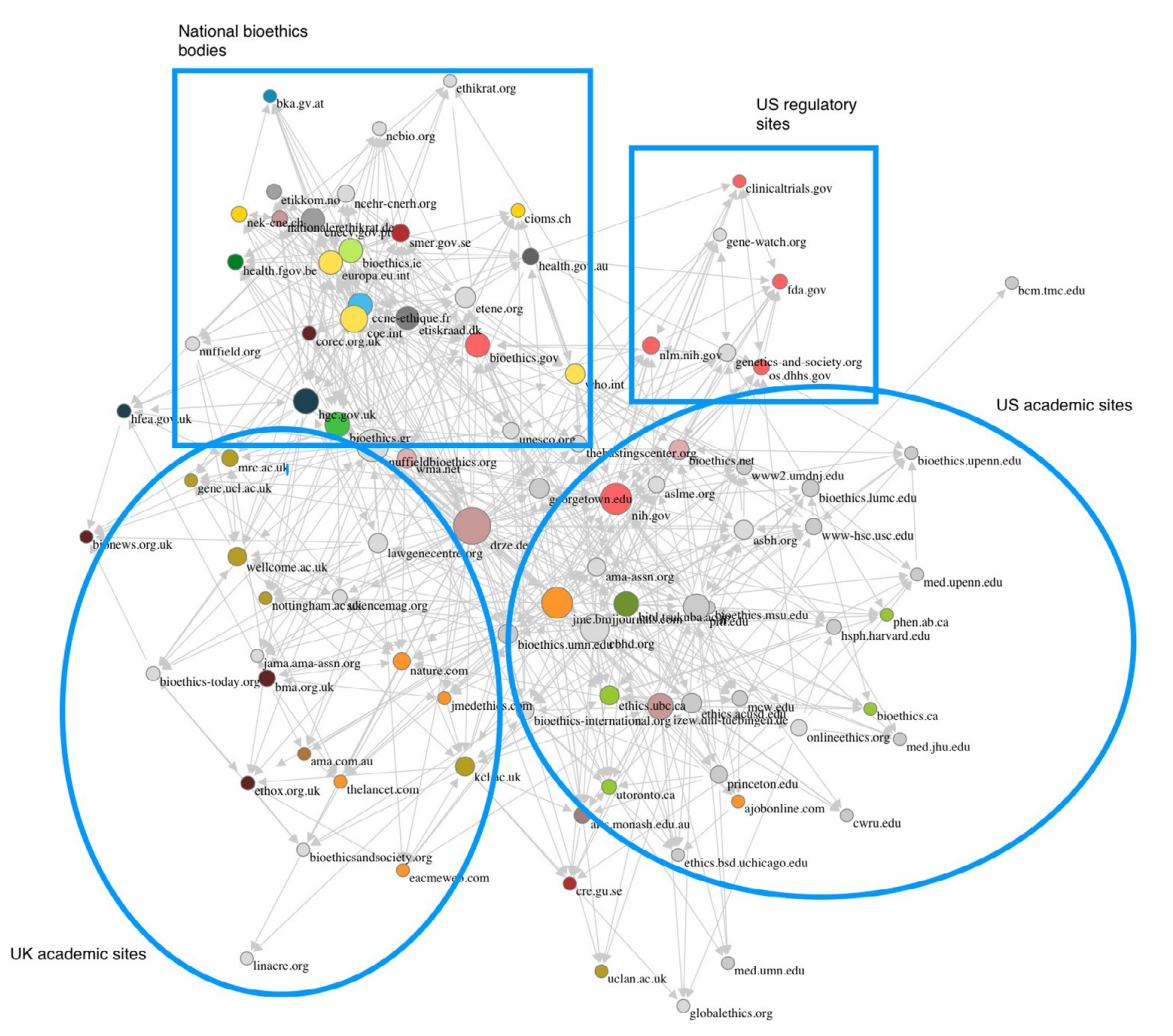
Web-linking according to in-links.

**Figure 2:**
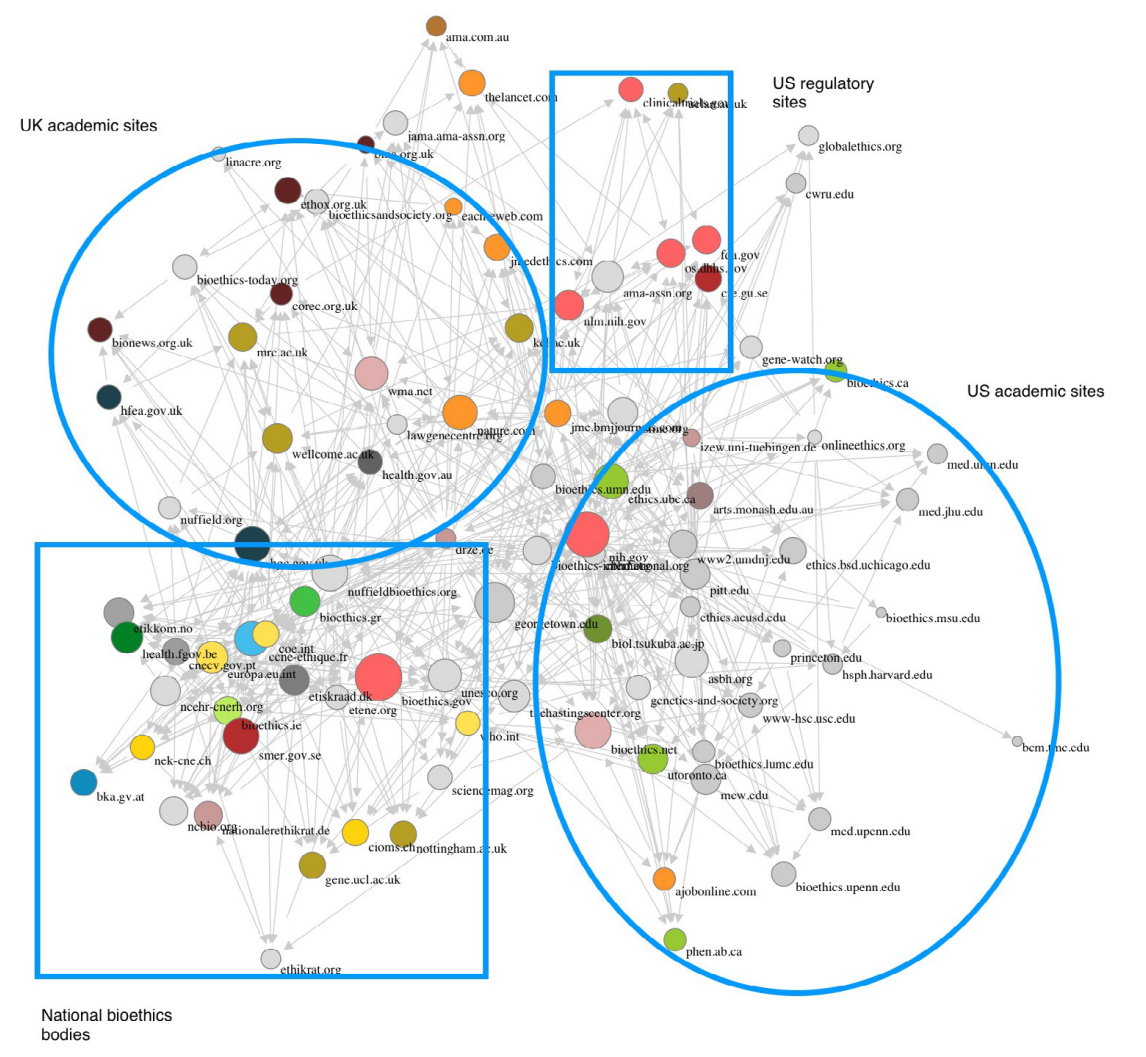
Web-linking according to in- and out-links.

Figures 1 and 2 show the results of this mapping (performed May 17, 2005). In order to test the stability of the results, we split our original list in two, simply taking every second website and running the IssueCrawler with each of these halved lists. This produced very similar maps to the ones produced by the complete list with identical clusters, indicating that the procedure reliably identifies the dominant or at least prominent websites and the main linking patterns in the bioethics field (data not shown).

In both website maps, it is possible to identify 4 main clusters: 1) a tightly linked cluster consisting mainly of websites of official, national bioethics bodies; 2) a loosely linked cluster of UK academic sites; 3) a loosely linked cluster of US academic sites; and 4) a small cluster of official US regulatory sites; interestingly, there was no significant clustering around either the Christian or other geographic categories. Interlinking between the clusters is limited and mainly through bioethics reference centers or a few other centers having very large link lists. The maps also clearly illustrate that a number of official websites are much more central when only in-links are taken into account than when both in- and out-links are counted, showing that whereas they are linked to, they themselves do not have large link lists.

### Analysis of citation patterns

When bioethicists write papers, they like other academics, are expected to provide references to the previous work in the field that they rely on. In principle, the reference for a given argument should be to the first author putting forward that idea (e.g., a reference for the 'non-identity' problem should cite Derek Parfit's *Reasons and Persons*). If bioethics were a unified global field, we should expect bioethicists in different countries to cite the same authors and sources. If they do not it is either because of an interest in different questions, because they read different journals, or because they are more likely to cite local authors even if these are actually not the originators of a given idea. There is no easy way of studying whether this is the case -detailed and in-depth citation studies are required [cf. [42-44]] – but there is a relatively simple way to investigate whether there is a correlation between the geographical origin of a paper and the location of those who cite that paper.

The ISI Web of Knowledge database (Science, Social Science, Arts and Humanities Citation indexes) includes almost 9000 journals across the full range of academic topics, including medicine, philosophy and sociology. For each paper indexed, the database also provides information about the papers in which the index paper has later been cited. In order to investigate whether US and non-US authors in bioethics have the same citation patterns (i.e., whether papers originating in the US are more likely to be cited by US authors than other papers), we identified all articles in the ISI Web of Knowledge database published between 1995–1997 with the keywords 'bioethics' or 'medical ethics'. We chose this timeframe because it is likely that most citations to these papers that will be made have already been made; the citation half-life of journal articles in the Web of Knowledge 'Medical Ethics' journal category is between 4.1 and 8.3 years (ISI Journal Citation Reports 2004 Science Edition). By choosing this starting point we ensure that difference in citation patterns found in our analysis will not be due to an immediacy effect, i.e., if there is a difference it is not because a paper is likely to be read and cited earlier by readers in the same geographical region than by readers in other regions. The specific features of the ISI Web of Knowledge database also limited the length of timeframe we could use since the standard version will only sort 300 papers according to number of citations.

The search gave an initial sample of 459 papers that had been published between 1995–1997 and had been cited at least once between its publication and June 2005. We split these into those papers where the lead author had a US address (228) and those where the lead author had a non-US address (231). Each subset was then sorted according to the number of times the paper had been cited in the literature. In each subset, the top 10 publications were chosen and the accuracy of the author address verified. The 735 papers citing one of these top 20 papers where then identified in the database and the bibliographic information downloaded to Endnote 6.02. Of the 735 papers, 505 cite one of the top 10 US publications, whereas 230 cite one of the top 10 non-US publications. We then divided these 735 publications into those with at least one US author and those with no US author. Table 1 shows the results.

**Table 1:** Citation of US and non-US Publications

	Cited publication is US publication	Cited publication is Non-US publication	Total
Citing publication has US author	260	84	344
Citing publication has no US author	245	146	391
Total	505	230	735

df = 1, Chi-Square = 14.21 p < 0.001

This analysis shows two things: 1) that the most cited US papers in the field are cited approximately twice as much as the most cited non-US papers, and 2) that papers citing US papers are significantly more likely to have at least one US author than papers citing non-US papers. Because the ISI Web of Knowledge only indexes the most important (and established) journals in each field, it is unlikely that these findings can be explained by differential availability (e.g., that US based authors are more likely to publish in US journals that are more likely to be available in US university libraries). The findings can be explained in two, not mutually exclusive ways. It could be the case that the topics of interest differ between bioethicists according to their geographical location. This would mean that papers from the same geographical region would be more likely to be relevant to subsequent analyses and therefore more likely to be cited. A rival explanation is simple citation bias, such that authors from one geographical region are simply more likely to cite authors from the same region. For our purposes, it does not matter which of these two explanations is correct, since they both, although in different ways, indicate that there is no unified global bioethics. The first explanation implies that bioethicists in different regions have different main interests, whereas the second implies something very close to parochialism.

### Online buying patterns

As a means of getting a more general sense of the type of public exposure and global uptake of bioethics literature, two 'bestseller' searches were conducted through the online bookstore Amazon, via their country specific websites, for the keyword 'bioethics'. The first search – conducted May 12, 2005 – collected a list of the top ten best selling English language 'bioethics' books on the Amazon country websites for the US (.com), the UK (.co.uk), Canada (.ca), Germany (.de) and France (.fr). By comparing individual book titles (see Additional file 2 for full dataset), it became apparent that there was actually relatively little overlap of books between countries; in fact, no book was in the top ten for more than two countries, a fact that would certainly be exacerbated had we included French or German language texts. To gain a sense of the type of books being purchased, each title was sorted into one of five rough categories (see Table 2):

**Table 2:** Top 10 'Bioethics' Books on Amazon, by Country

	US	Canada	UK	Germany	France
CT (course text)	5	3	3	5	2
P (professional)	1	1	1	0	0
R (religion)	1	0	0	1	1
ST (specific topic)	2	3	6	2	2
TH (theory)	1	3	0	2	5

For full data, see Additional file 2

1. Course text (CT): introductory bioethics textbooks, usually collections of 'classic' articles or case studies of major issues;

2. Professional (P): clinical or professional ethics training guides written for health care professionals;

3. Religion (R): texts taking particular religious perspectives, usually Christian, on various bioethics topics;

4. Specific topic (ST): texts focused on topics such as stem cells, euthanasia, genetics;

5. Theory (TH): texts examining the field of bioethics, its theories, histories, critiques, etc.

Interestingly, while one might have assumed that Canada and the US would have similar profiles – because of their geographic proximity, close economic ties, and shared history in the development of bioethics as a profession – Canada actually more closely resembles the UK in the popularity of various types of bioethics books, while the German profile is very similar to that of the US. Given the long history of bioethics education in the US, it is not surprising that 5 of the 10 bestsellers are introductory course texts, though one might have expected greater prominence of texts on specific topics, theory or religious perspectives; this is more apparent in the top 50 bestsellers (see Table 3). Due to our focus on English language bioethics books, we can only speculate as to the implications of the German and French profiles; from the number of English books listed and cursory searches in French and German, we can safely assume that German and French audiences are reading books in both English and their own languages. The German profile does, for instance, seem to indicate a quite interesting pattern of reception of English-language bioethics with a very strong representation of books taking radical positions.

**Table 3:** Top 50 'Bioethics' Books on Amazon, US & UK

	US	UK
CT (course text)	17	12
P (professional)	2	2
R (religion)	12	9
ST (specific topic)	9	22
TH (theory)	10	5

For full data, see Additional file 3

A second search conducted June 7, 2005 compared the top 50 bestsellers in the US (out of a total of 818 books) and the UK (out of a total of 798 books). The same five categories used in the first search were then applied to the results. As shown in Table 3, there was a dramatic difference in the category of book popular in each country. The US top 50 was dominated by course texts, while for the UK it was books on specific topics that were most popular. Also, as mentioned previously, the US top 50 contains many more books on specific topics and religious perspectives than either the US top 10 or the UK top 50. (It should be noted that in the three week interval between the two searches, i.e., Tables 2 and 3 respectively, the ranking of bestsellers had changed).

This search approach has obvious limitations. The data presented will, to some extent, hide the true numbers of country specific buyers. A consumer in the UK or Canada, for example, can still buy through the main (and thus more comprehensive) Amazon.com site, and may in fact do so because some books are available on this site and not another. Further, given their proximity to the US and access to cheaper shipping fees, Canadians may be more likely to purchase books from Amazon.com than would their European counterparts. More generally, the Amazon bestseller search is unlikely to tell us anything specific about the buying habits of students or academics, or uptake of bioethics texts for academic courses, as university libraries tend to rely on mainstream booksellers both for stocking libraries and supplying course texts. Further, these results will be in flux continuously, for as more people buy books, the bestseller rankings will change. Nevertheless, this brief and time delimited Amazon search tells us something about the behavior of individuals in the general public, which necessarily includes students, academics and professionals studying or working in the field of bioethics. What is apparent from the results presented in Tables 2 and 3 is that, perhaps not surprisingly, the buying behavior varies substantially by country.

## Conclusion

All three of our exploratory studies of behavioral patterns in the bioethics field support the position that there is in fact no unified global field of bioethics. It seems that, even in English-speaking countries, bioethicists do not link to each other's websites as much as would be expected, do not cite each other as much as would be expected, and do not converge on the same books as much as would be expected if bioethics were truly a 'global' field. These studies furthermore indicate that there are at least two types of disjunctions: 1) a geographical disjunction, or many depending on one's interpretation of the data, and 2) a disjunction between official bioethics and academic bioethics visible in the web linking analysis. If we had broadened our study to include non-English bioethics, we would undoubtedly have also found a linguistic disjunction. The most popular French and German bioethics books sold by Amazon in France and Germany are, for instance, even more dissimilar to the US or UK favorites in both topics and authors.

Recently Borry, Schotsmans and Dierickx have studied publication patterns in bioethics journals and found that,

While a lot of peer reviewed journals in the field of bioethics profile themselves as international journals, they certainly do not live up to what one would expect from an 'international' journal. The fact that English speaking countries, and to a larger extent American authors, dominate the international journals in the field of bioethics is a clear geographic bias towards the bioethical discussions that are going on in these journals [45].

Borry et al's findings are thus consistent with our own. Our studies only sampled five countries in North America and Europe, so we obviously cannot pretend to draw concrete conclusions about the state and practice of bioethics around the world. Nevertheless, given the significant lack of cohesion within our small sample of 'Western' countries, we can reasonably conclude that that patterns of 'bioethics behavior' in, say Latin America, Asia or Africa would further reinforce these differences.

Let us assume that our analysis is correct and that there really is no unified global field of bioethics, and that bioethicists in different regions differ in the way they approach and practice bioethics. Is that a problem? Well it is a problem if the only reason is parochialism. Parochialism may be understandable to some degree because specific concerns are to some extent local, but new and better communication possibilities (e.g., email, web chat rooms, open access journals, free/cheap Internet telephony, lower airfares) ought by now to have worked against this geographical isolation. Parochialism is also a potential problem for those outside the field who might not understand that bioethics is a cluster concept, especially if those outsiders (e.g., members of the general public, other academics or professionals, policy makers, politicians) seek to use bioethics as a source of moral truth or as a 'whipping boy'. But these regional differences are probably much less of a problem within the field – as any attentive insider would have noticed long ago, bioethics comes in many not always mutually understandable dialects.

## Competing interests

The author(s) declare that they have no competing interests.

## Authors' contributions

SH conducted the IssueCrawler and Citations analyses. BWJ conducted the Amazon searches. Both authors were equally involved in the conceptualization, production and editing, and read and approved the final manuscript.

## Pre-publication history

The pre-publication history for this paper can be accessed here:



## Supplementary Material

Additional file 1 Starting List of Websites for IssueCrawler Web Crawl. Click here for file

Additional file 2 Top 10 'Bioethics' Books on Amazon, By Country. Click here for file

Additional file 3 Top 50 'Bioethics' Books on Amazon, US & UK. Click here for file
