# Supplementary material for: Global bioethics – myth or reality?
Source: BMC Med Ethics. 2006 Sep 11;7:10. doi: 10.1186/1472-6939-7-10 (PMC1592295; doi:10.1186/1472-6939-7-10)
Supplement: Additional file 1 — Starting List of Websites for IssueCrawler Web Crawl. [file 1472-6939-7-10-S1.doc]

**Starting List of Websites for IssueCrawler Web Crawl**

***General link lists***

http://www.eacmeweb.com/all-members

http://www.aem-online.de/main.htm

http://jme.bmjjournals.com/misc/links.shtml

http://www.biol.tsukuba.ac.jp/~macer/Info.html

http://www.ethicsweb.ca/

http://www.genethics.ca/people.html

http://bioethicsweb.ac.uk/

http://www.mic.ki.se/Diseases/K01.316.html

http://www.bioethics.ntnu.no/index.php?id=lenker

http://www.aslme.org/links/index.php

***Official organizations***

http://www.unesco.org/shs/bioethics

http://www.who.int/ethics/en/

http://europa.eu.int/comm/european_group_ethics/liens_en.htm

http://www.etiskraad.dk/sw408.asp

http://www.ccne-ethique.fr/english/start.htm

http://www.nuffieldbioethics.org/go/furtherinformation/links.html

http://www.nih.gov/sigs/bioethics/organizations.html

***Christian***

http://www.linacre.org/frames.html

http://www.lindeboominstituut.nl/

http://www.wheaton.edu/CACE/links/linksmain.htm

http://www.cbhd.org/links/index.html

http://www.parkridgecenter.org/Page1266.html

***France***

http://www.chu-rouen.fr/ssf/bioethfr.html

http://cem.icl-lille.fr/

http://infodoc.inserm.fr/ethique/ethique.nsf/

http://www.ap-hm.fr/ethiq/fr/site/accueil.asp

http://www.espace-ethique.org

***Germany***

http://www.drze.de/links

http://www.ruhr-uni-bochum.de/zme/verweise.htm

http://www.kritischebioethik.de/deutschland_links.html

***United Kingdom***

http://www.shef.ac.uk/b/bioethics-today/FSresearch.htm

http://www.practicalethics.ox.ac.uk/ethics_resources.html

http://www.nottingham.ac.uk/bioethics/theory_pages/links.htm

http://www.bioethics.ac.uk/links.html

http://www.ccels.cardiff.ac.uk/contacts/index.html

***Italy and Spain***

http://www.bioetica-vssp.it/link.html

http://www.consultadibioetica.org/generale/siti.HTM

http://www.bioeticaweb.com/

http://www.bioetica.org/

http://bioetica.bvsalud.org/html/es/home.html

http://www.ub.es/fildt/eenlaces.htm

***United States***

http://www.princeton.edu/~bioethic/resources/links.html

http://wings.buffalo.edu/faculty/research/bioethics/other.html

http://www.georgetown.edu/research/nrcbl/orgs.htm

http://ethics.bsd.uchicago.edu/resources.html

http://csmeh.mc.duke.edu/

http://www.pitt.edu/~bioethic/links.htm

http://www.csuohio.edu/bioethics/links.htm
