# Supplementary material for: Global bioethics – myth or reality?
Source: BMC Med Ethics. 2006 Sep 11;7:10. doi: 10.1186/1472-6939-7-10 (PMC1592295; doi:10.1186/1472-6939-7-10)
Supplement: Additional file 2 — Top 10 'Bioethics' Books on Amazon, By Country. [file 1472-6939-7-10-S2.doc]

**Top 10 ‘Bioethics’ Books on Amazon, By Country**

|  | **US (.com)** | **Canada (.ca)** | **UK (.co.uk)** | **Germany (.de)** | **France (.fr)** |
| --- | --- | --- | --- | --- | --- |
| 1 | *In the Wake of Terror : Medicine and Morality in a Time of Crisis*, Jonathan D. Moreno (2003) | *The Virtues in Medical Practice,* Edmund D. Pellegrino (1993) | *The Human Embryonic Stem Cell Debate: Science, Ethics and Public Policy* Suzanne Holland, et al. (2001) | *A Companion to Bioethics*, Helga Kuhse, Peter Singer (2001) | *Strangers at the Bedside: A History of How Law and Bioethics Transformed Medical Decision Making*, David J. Rothman (2003) |
| 2 | *The Human Embryonic Stem Cell Debate: Science, Ethics, and Public Policy*, Suzanne Holland et al (2001) | *Principles of Biomedical Ethics*,  Tom L. Beauchamp, James F. Childress (2001) | *Beyond Therapy: Biotechnology and the Pursuit of Happiness*,  Presidents Council on Bioethics (2004) | *Textbook of Healthcare Ethics*, Erich H. Loewy (2004) | *Bioethics in a European Perspective*, Henk A.M.J. Ten Have, Bert Gordijn (2001) |
| 3 | *Life, Liberty and the Defense of Dignity: The Challenge for Bioethics*, Leon Kass (2004) | *Sourcebook in Bioethics*, Albert R. Jonsen et al (2002) | *Bioethics: An Anthology*, Helga Kuhse, Peter Singer (1999) | *Bioethics and Moral Content,* H. Tristram Engelhardt (2003) | *Bioethics*, Peter Singer, Helga Kuhse (2005) |
| 4 | *Bioethics Mediation: A Guide to Shaping Shared Solutions*, Nancy N. Dubler, Carol B. Liebman (2003) | *The Second Tree: An Investigation Into Stem Cells, Cloning, and the Quests for Immortality*, Elaine Dewar (2005) | *Bioethics (Oxford Readings in Philosophy)*, John Harris (2001) | *Hindu Bioethics for the Twenty-First Century*, S. Cromwell Crawford (2003) | *Bioethics*, Peter Singer, Helga Kuhse (2005) |
| 5 | *Contemporary Issues in Bioethics*, Tom L. Beauchamp, LeRoy Walters (2002) | *Clones, Genes and Immortality: Ethics and Genetics*,  John Harris (1998) | *Animal Rights: A Very Short Introduction*, David DeGrazia (2002) | *Meta Medical Ethics: The Philosophical Foundations of Bioethics*, Michael A. Grodin (2001) | *Genetics: Science, Ethics, and Public Policy*, Thomas A. Shannon (2005) |
| 6 | *Bioethics: A Primer For Christians*, Gilbert Meilaender (2004) | *Biomedicine and the Human Condition: Challenges, Risks and Rewards*, Michael Sargent (Cambridge UP, 2005) | *Autonomy and Trust in Bioethics*, Onora O'Neill (Cambridge UP, 2002) | *Animal Liberation*, Peter Singer, Susan Reich (1990) | *The Law of Ethics and Medical Research: International Bioethics and Human Rights*, Aurora Plomer (2005) |
| 7 | *The Basics of Bioethics*, Robert M. Veatch (2002) | *Stories Matter: The Role of Narrative in Medical Ethics*, Rita Charon, Martha Montello (2002) | *Clones, Genes and Immortality: Ethics and Genetics*, John Harris (1998) | *Bioethics*, John Harris (2001) | *American Bioethics: Crossing Human Rights and Health Law Boundaries*, George J. Annas (2005) |
| 8 | *Cases in Bioethics: Selections from the Hastings Center Report,* Bette-Jane Crigger (1998) | *Bioethics at the bedside: A clinician's guide*,  Peter Singer (1999) | *A Companion to Bioethics*, Helga Kuhse, Peter Singer (2001) | *Ten Trusts,* Jane Goodall, Marc Bekoff (2003) | *Bioethics: A Primer For Christians*, Gilbert Meilaender (2005) |
| 9 | *Bioethics: An Anthology*, Helga Kuhse, Peter Singer (1999) | *The National Bioethics Advisory Commission*, Elisa Eiseman (2004) | *Biomedicine and the Human Condition: Challenges, Risks and Rewards*, Michael Sargent (2005) | *Life, Liberty and the Defense of Dignity: The Challenge for Bioethics*, Leon. Kass (2004) | *The Second Tree: Stem Cells, Clones, Chimeras, and Quests for Immortality*, Elaine Dewar (2005) |
| 10 | *Outcome Uncertain: Cases and Contexts in Bioethics*,  Ronald Munson et al (2002) | *Foundations of Bioethics*, H. Tristram Engelhardt (1996) | *Bioethics for Scientists*, John A. Bryant et al (2002) | *Being Human*, Leon Kass (2004) | *Bioethics: A Culture War*, Nicholas C. Lund-Molfese, Michael L. Kelly (2004) |

Search conducted May 12, 2005, for English language books top 10, sorted by Bestselling. Colours identify matching titles.
