# Supplementary material for: Global bioethics – myth or reality?
Source: BMC Med Ethics. 2006 Sep 11;7:10. doi: 10.1186/1472-6939-7-10 (PMC1592295; doi:10.1186/1472-6939-7-10)
Supplement: Additional file 3 — Top 50 'Bioethics' Books on Amazon, US & UK. [file 1472-6939-7-10-S3.doc]

**Top 50 ‘Bioethics’ Books on Amazon, US & UK**

|  | US (.com) of 818 |  | UK (.co.uk) of 798 |  |
| --- | --- | --- | --- | --- |
| 1 | *Bioethics: A Primer For Christians*, Gilbert Meilaender (2004) | R | *Autonomy and Trust in Bioethics*, Onora O'Neill (Cambridge UP, 2002) | ST |
| 2 | *The Human Embryonic Stem Cell Debate: Science, Ethics, and Public Policy*, Suzanne Holland et al (2001) | ST | *Bioethics: An Anthology*, Helga Kuhse, Peter Singer (1999) | CT |
| 3 | *Contemporary Issues in Bioethics*, Tom L. Beauchamp, LeRoy Walters (2002) | CT | *A Companion to Bioethics*, Helga Kuhse, Peter Singer (2001) | CT |
| 4 | *Bioethics: A Return to Fundamentals*,  Bernard Gert, et al (1997) | CT | *Bioethics*, John Harris (2001) | CT |
| 5 | *Pricing Life: Why It's Time for Health Care Rationing*,  Peter A. Ubel (2001) | ST | *Animal Rights: A Very Short Introduction*, David DeGrazia (2002) | ST |
| 6 | *Bioethics: An Anthology*, Helga Kuhse, Peter Singer (1999) | CT | *Clones, Genes and Immortality: Ethics and Genetics*, John Harris (1998) | ST |
| 7 | *Life, Liberty and the Defense of Dignity: The Challenge for Bioethics*, Leon Kass (2004) | TH | *Stories Matter: The Role of Narrative in Medical Ethics*, Rita Charon, Martha Montello (2002) | ST |
| 8 | *Catholic Bioethics and the Gift of Human Life,* William E. May (2000) | R | *Key Issues in Bioethics*, Ralph Levinson, Michael Reiss (2003) | CT |
| 9 | *Cases in Bioethics: Selections from the Hastings Center Report,* Bette-Jane Crigger (1998) | CT | *Animal Welfare: A Cool Eye Towards Eden,* John Webster (1995) | ST |
| 10 | *A Companion to Bioethics*, Helga Kuhse, Peter Singer (2001) | CT | *The Law and Ethics of Medical Research: International Bioethics and Human Rights*, Aurora Plomer (2005) | ST |
| 11 | *The Basics of Bioethics*, Robert M. Veatch (2002) | CT | *The Human Embryonic Stem Cell Debate: Science, Ethics and Public Policy* Suzanne Holland, et al. (2001) | ST |
| 12 | *Bioethics Mediation: A Guide to Shaping Shared Solutions*, Nancy N. Dubler, Carol B. Liebman (2004) | P | *Biomedicine and the Human Condition: Challenges, Risks and Rewards*, Michael Sargent (Cambridge UP, 2005) | CT |
| 13 | *Case Studies in Biomedical Research Ethics*, Timothy F. Murphy (2004) | CT | *What Genes Can't Do,* Lenny Moss (2004) | ST |
| 14 | *Medical Law, Ethics, and Bioethics for Ambulatory Care*, Marcia A. Lewis, Carol D. Tamparo (2002) | ST | *Bioethics for Scientists*, John A. Bryant et al (2002) | P |
| 15 | *Human Cloning and Human Dignity: The Report of the President's Council on Bioethics*, Leon R. Kass (2002) | ST | *Bioethics: A Philosophical Introduction*, Stephen Holland (2003) | CT |
| 16 | *An Introduction to Bioethics*, Thomas A. Shannon (1996) | CT | *Ten Trusts: What We Must Do to Care For the Animals We Love*, Jane Goodall, Marc Bekoff (2004) | ST |
| 17 | *Strangers at the Bedside: A History of How Law and Bioethics Transformed Medical Decision Making*, David J. Rothman (2003) | TH | *On Being Human: Where Ethics, Medicine and Spirituality Converge*, Daisaku Ikeda, et al (2003) | R |
| 18 | *Encyclopedia of Bioethics*, Stephen G. Post (2003) | TH | *Case Studies in Biomedical Research Ethics*, Timothy F. Murphy (2004) | CT |
| 19 | *Genetics and Life Insurance: Medical Underwriting and Social Policy*, Mark A. Rothstein (2004) | ST | *Beyond Therapy: Biotechnology and the Pursuit of Happiness*,  Presidents Council on Bioethics (2004) | ST |
| 20 | *The Reproduction Revolution: A Christian Appraisal of Sexuality, Reproductive Technologies, and the Family*, Center for Bioethics and Human Dignity, et al (2000) | R | *Human Dignity in Bioethics and Biolaw*, Deryck Beyleveld, Roger Brownsword (2001) | CT |
| 21 | *Law and Bioethics: An Introduction*, Jerry Menikoff (2002) | CT | *Theological Issues in Bioethics: An Introduction with Readings*,  Neil Messer (2002) | R |
| 22 | *Cutting-Edge Bioethics: A Christian Exploration of Technologies and Trends*, John Frederic Kilner, et al (2002) | R | *The Human Cloning Debate*, Glenn McGlee (2004) | ST |
| 23 | *Hindu Bioethics for the Twenty-First Century*, S. Cromwell Crawford (2003) | R | *The Lives to Come: the Genetic Revolution and Human Possibilities*, Philip Kitcher (1997) | ST |
| 24 | *Foundations of Bioethics*, H. Tristram Engelhardt (1996) | CT | *Contemporary Issues in Bioethics*, Tom L. Beauchamp, LeRoy Walters (2002) | CT |
| 25 | *Considering Religious Traditions in Bioethics: Christian and Jewish Voices,* Mary Jo Iozzio (2001) | R | *Genetic Dilemmas: Reproductive Technologies, Parental Choices and Children's Futures*, Dena S. Davis (2001) | ST |
| 26 | *Is Human Nature Obsolete? : Genetics, Bioengineering, and the Future of the Human Condition*, Harold W. Baillie, Timothy K. Casey (2004) | ST | *Catholic Bioethics and the Gift of Human Life,* William E. May (2000) | R |
| 27 | *American Bioethics: Crossing Human Rights and Health Law Boundaries*, George J. Annas (2004) | CT | *Biomedical Ethics*, Thomas A. Mappes, David DeGrazia (2000) | CT |
| 28 | *Notes from a Narrow Ridge: Religion and Bioethics*, Dena S. Davis, Laurie Zoloth (1999) | R | *Bioethics: A Primer For Christians*, Gilbert Meilaender (1996) | R |
| 29 | *Introduction to Jewish and Catholic Bioethics: A Comparative Analysis*, Aaron L. Mackler (2003) | R | *Life, Liberty and the Defense of Dignity: The Challenge for Bioethics*, Leon Kass (2004) | TH |
| 30 | *Cases in Bioethics: Selections from the Hastings Center Report,* (1989) | CT | *The Ethics of Human Cloning,* Leon R. Kass, James Q. Wilson (1998) | ST |
| 31 | *God in the Laboratory: Equipping Christians to Deal With Issues in Bioethics*, Al Truesdale (2000) | R | *Culture of Death: The Assault on Medical Ethics in America*, Wesley J. Smith (2000) | ST |
| 32 | *Life's Worth: The Case Against Assisted Suicide*, Arthur J. Dyck (2002) | ST | *Buddhism and Bioethics*, Damien Keown (2001) | R |
| 33 | *End-of-Life Decision Making : A Cross-National Study*, Robert H. Blank, Janna C. Merrick (2005) | ST | *DNA and the Criminal Justice System : The Technology of Justice*, David Lazer (2004) | ST |
| 34 | *Bioengagement: Making a Christian Difference Through Bioethics Today*, Nigel M. De S. Cameron, et al (2000) | R | *Bioethics: A Nursing Perspective*, Megan-Jane Johnstone (2004) | P |
| 35 | *Bioethics: A Christian Approach in a Pluralistic Age*, Scott B. Rae, et al (1999) | R | *Dubious Equalities and Embodied Differences: Cultural Studies on Cosmetic Surgery*, Kathy Davis (2003) | ST |
| 36 | *DNA and the Criminal Justice System : The Technology of Justice*, David Lazer (2004) | ST | *The Green Halo: A Bird's-eye View of Ecological Ethics,* Erazim Kohak (1999) | ST |
| 37 | *Beginning Bioethics: A Text with Integrated Readings*, Aaron Ridley (1997) | CT | *The Dream of the Perfect Child*, Joan Rothschild (2005) | ST |
| 38 | *Pragmatic Bioethics: Second Edition*, Glenn McGee (2003) | TH | *Deciding for Others: The Ethics of Surrogate Decision Making*, Allen E. Buchanan, Dan W. Brock (1990) | ST |
| 39 | *Feminist Approaches to Bioethics: Theoretical Reflection and Practical Applications*, Rosemarie Tong (1997) | TH | *The Biology of Moral Systems*, Richard D. Alexander (1987) | ST |
| 40 | *Do We Still Need Doctors?: A Physician's Personal Account of Practicing Medicine Today*, John D., MD Lantos (1999) | P | *Biology and the Foundations of Ethics*, Jane Maienschein, Michael Ruse (1999) | CT |
| 41 | *Bioethics: A Philosophical Introduction*, Stephen Holland (2003) | CT | *Strangers at the Bedside: A History of How Law and Bioethics Transformed Medical Decision Making*, David J. Rothman (2003) | TH |
| 42 | *Readings in Comparative Health Law and Bioethics*, Timothy Stoltzfus Jost (2001) | CT | *Playing God!: Human Genetic Engineering and the Rationalization of Public Bioethical Debate 1959-1995*, John H. Evans (2002) | ST |
| 43 | Bioethics and the Common Good, Lisa Sowle Cahill (2004) | TH | *Does God Need Our Help?: Cloning, Assisted Suicide, & Other Challenges in Bioethics*, John Frederic Kilner, et al (2003) | R |
| 44 | *More Humane Medicine: A Liberal Catholic Bioethics*, James F. Drane (2003) | R | *The Foundations of Christian Bioethics*, H. Tristram Engelhardt Jr (2000) | R |
| 45 | *Bioethics & Law: Cases and Materials*, Michael H. Shapiro, et al (2002) | CT | *Christians and Bioethics*, Fraser Watts (2000) | R |
| 46 | *Globalizing Feminist Bioethics: Crosscultural Perspectives*, Rosemarie Tong, et al (2000) | TH | *The Birth of Bioethics*, Albert R. Jonsen (2003) | TH |
| 47 | *Fragmentation and Consensus: Communitarian and Casuist Bioethics*,  Mark G. Kuczewski (1999) | TH | *The Elimination of Morality: Reflections on Utilitarianism and Bioethics*, Anne Maclean (1993) | TH |
| 48 | *Bioethics: Volume 19, Part 2*, Ellen Frankel Paul, et al (2002) | CT | *Stories and Their Limits: Narrative Approaches to Bioethics*, Hilde Lindemann Nelson (1998) | TH |
| 49 | *Embodying Bioethics: Recent Feminist Advances : Recent Feminist Advances*, Anne Donchin (1999) | TH | *Essays on Bioethics*, R. M. Hare (1996) | CT |
| 50 | *Taking Issue: Pluralism And Casuistry In Bioethics,* Baruch A. Brody (2004) | TH | *The Sacred Gift of Life: Orthodox Christianity and Bioethics,* John Breck (1999) | R |

Search conducted June 7, 2005, for English language books, sorted by Bestselling.
